# Negligible Senescence

**DOI:** 10.3389/fgene.2012.00151

**Published:** 2012-08-28

**Authors:** Daniel E. Martínez

**Affiliations:** ^1^Department of Biology, Pomona CollegeClaremont, CA, USA

In the development of a “new evolutionary genetics of aging” there is one issue that, in my opinion, requires additional attention: negligible senescence. Trees with longevities of hundreds of years or small invertebrates, like the hydra, that appear immortal seem to defy traditional theories of aging. Can we imagine a scenario in which Hamiltonian forces of natural selection never decline? Can some species maintain an age-independent adaptive tuning? Are particular physiologies more “permissive” than others to the evolution of better adaptive tuning? The acknowledgment that late-life plateaus in mortality and fecundity are real phenomena rather than artifacts fostered important progress, both theoretical and experimental. At first sight negligible aging, like cessation of aging, does not seem to fit neatly under Hamilton’s theory of a decline of the force of natural selection with increasing age. Unless we do not believe that negligible senescence is real, it seems that we should seek a better explanation for it.

Understandably, the experimental aging field has been mainly focused on the study of short-lived animal and plant models. Given our own limited lifespan, the study of species with negligible aging is likely to demand very creative approaches. Evolutionary biology should provide the framework to guide that research.

